# Aseptic presentation of interventricular septal abscess with progressive heart block: a case report

**DOI:** 10.1186/s13256-023-03846-9

**Published:** 2023-04-10

**Authors:** Pramukh Arun Kumar, Boskey Patel, Mahati Dasari, Sumukh Arun Kumar, Neeta Shah, Douglas Laidlaw

**Affiliations:** 1 Department of Internal Medicine, Saint Vincent Hospital, 123 Summer Street, Worcester, MA 01608 USA; 2 Division of Cardiovascular Medicine, Lahey Hospital and Medical Center, Burlington, MA USA; 3 Division of Cardiovascular Medicine, Saint Vincent Hospital, Worcester, MA USA

**Keywords:** Complete heart block, Interventricular septal abscess, Intracardiac abscess, Infective endocarditis, Aortic root abscess

## Abstract

**Background:**

Infective endocarditis can progress to an intracardiac abscess in 20% to 30% of cases, with interventricular septal abscess (IVSA) being one of the rare complications usually presenting with sepsis. We present a case of IVSA presenting with a new-onset second-degree heart block, which rapidly progressed to a complete heart block.

**Case presentation:**

A 80-year-old Caucasian female with a past medical history of hypertension and hyperlipidemia presented with exertional chest pain, lightheadedness, and shortness of breath with telemetry and electrocardiogram revealing persistent Mobitz type II second degree atrioventricular block. The rest of the vitals were normal. As she was being planned for a pacemaker placement, she spiked a temperature of 103F. Blood cultures grew methicillin-sensitive Staphylococcus aureus, and appropriate antibiotics were initiated. Transthoracic echocardiogram was grossly normal. However, transesophageal echocardiogram revealed a heterogeneous extension of an echodensity from the aortic root, along the aorto-mitral cushion and into the interventricular septum, indicating an interventricular septal abscess. Her course was complicated by altered mental status, with computed tomography of the brain revealing hypodense regions in the left lentiform nucleus and anterior caudate nucleus representing acute/subacute stroke. Surgery was deferred as she was deemed a poor candidate. She succumbed to her illness on day 6 of hospitalization.

**Conclusion:**

Intracardiac abscesses should be considered a possible initial differential in patients with progressive heart block despite aseptic presentation and no risk factors.

## Background

Infective endocarditis (IE) presents a myriad of clinical features that depend on the causative organism, its virulence, duration of bacteremia, and presence of implantable cardiac devices [1]. Adding to this clinical complexity, any microorganism is known to cause IE, as described in various case reports [2, 3]. Considering these evolving clinical findings, there is a concern for the delay in diagnosis leading to worse outcomes. Among these, intracardiac abscesses are life-threatening complications of IE, which hold high in-hospital mortality despite the availability of newer antimicrobials and modern surgical techniques [4]. Here is a case of intracardiac abscess with an underreported presentation.

## Case presentation

An 80-year-old Caucasian female with a past medical history of hypertension and hyperlipidemia presented with exertional midsternal chest pain that was predominantly pressure-like, 5/10 in intensity, and short-lasting. There was no associated shortness of breath, sweating, or palpitations. On the same night, she had an episode of lightheadedness associated with shortness of breath as she tried to get up from bed. This lightheadedness was not associated with fever, chills, malaise, myalgia, night sweats, loss of consciousness, or dizziness. She had no history of intravenous drug use, prior/recent dental procedures, prosthetic valves, rheumatic heart disease, or immunocompromised states. Vital signs included a temperature of 98.7 F, heart rate of 38 beats per minute, blood pressure of 122/58 mmHg, and oxygen saturation of 98% on room air. Orthostatic vitals were normal. Cardiac examination revealed bradycardia, normal S1 and S2, and no audible murmurs. The remainder of the examination was unremarkable except for trace bilateral lower extremity edema.

**Fig. 1:**
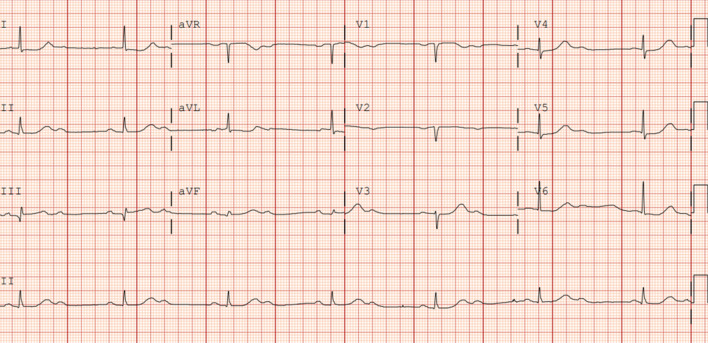
Electrocardiogram showing sinus rhythm with 2:1 atrioventricular block

**Fig. 2:**
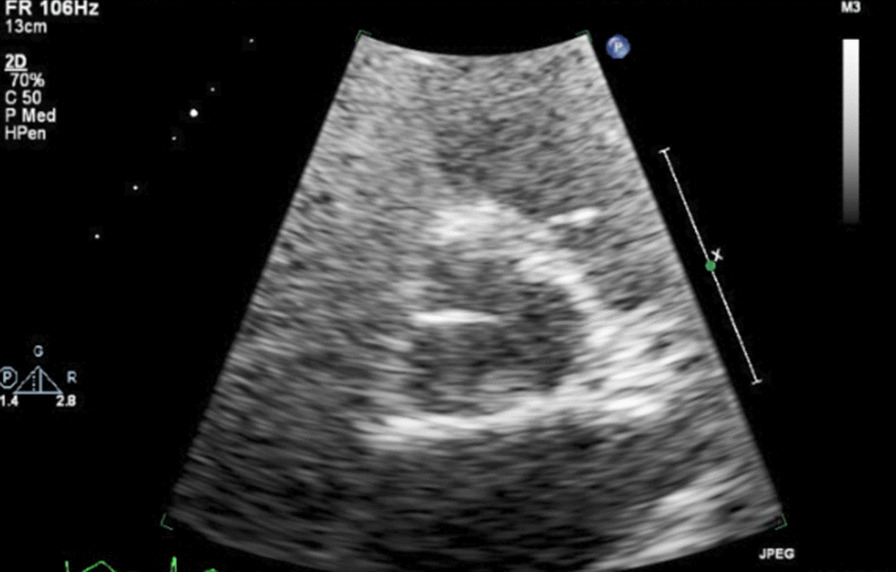
Transthoracic echocardiogram in parasternal short axis view showing aortic valve annular calcification

**Fig. 3:**
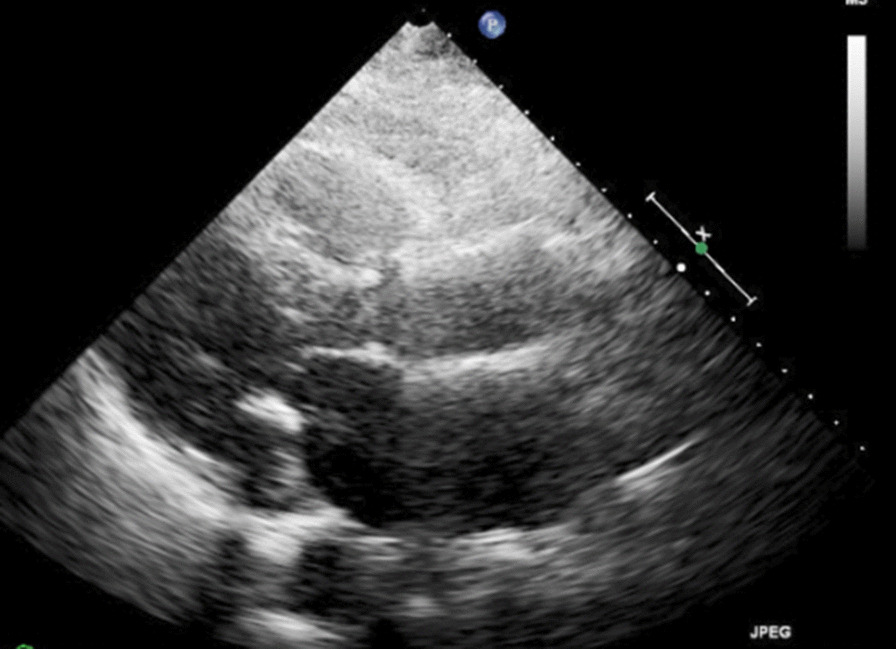
Transthoracic echocardiogram in parasternal long axis view showing calcified appearance of mitral and aortic annuli with nonspecific thickening of the interventricular septum

Initial laboratory workup, including complete blood count, complete metabolic panel, and thyroid panel, was significant for mild leukocytosis of 11,300 cells per mm^3^ (normal reference range: 3900–11,000 cells per mm^3^). Cardiac biomarkers showed normal troponin and elevated brain natriuretic peptide (BNP) pro of 13,443 pg/mL (normal reference range: less than 125 pg/mL). Chest x-ray (CXR) showed no acute cardiopulmonary process. Electrocardiogram (EKG) showed sinus rhythm with 2:1 atrioventricular block on admission (Fig. 1), but telemetry showed intermittent complete heart block. Transthoracic echocardiogram (TTE) images were suboptimal due to poor windows and motion artifacts. However, it revealed normal left ventricular (LV) systolic function with mild concentric LV hypertrophy and an ejection fraction of 70%. There were no apparent valvular abnormalities (Figs. 2, 3).

**Fig. 4:**
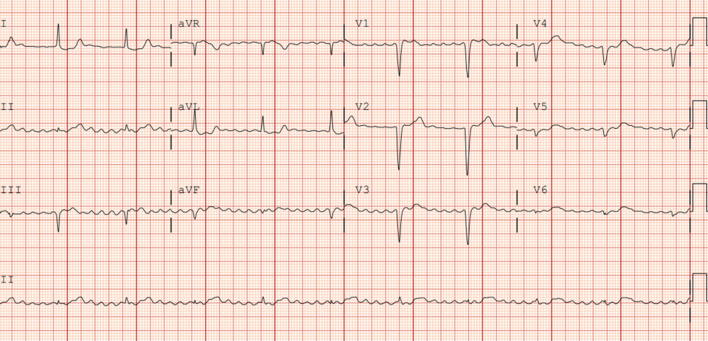
Day 4 Electrocardiogram showing atrial fibrillation with rate regularization consistent with complete heart block

On the day of admission, clonidine and atenolol (home antihypertensives) were discontinued due to suspicion of medication-induced conduction abnormality. Despite this, telemetry showed persistent high-degree heart block with a heart rate in the 40 s. The rest of her vitals remained stable. Hence, she was scheduled for a permanent pacemaker implantation for a presumptive diagnosis of senile high-degree heart block. On day 3 of admission, before the scheduled procedure, she became acutely hypoxic to 85%, requiring a non-rebreather mask to maintain adequate oxygen saturation. CXR revealed pulmonary vascular congestion, which was appropriately treated with diuretics following which she improved to saturate well on 4 L of oxygen via nasal cannula. However, she also spiked a single elevated temperature of 103F. Blood cultures grew gram-positive cocci in clusters in 4/4 bottles. Empirical intravenous vancomycin was started. On day 4, she developed new onset slurred speech, right-sided hemiparesis, and altered mental status. Physical examination revealed no signs or sequelae of systemic embolization, and her skin was warm and well-perfused. Computed tomography of the brain revealed hypodense regions in the left lentiform nucleus and anterior caudate nucleus representing acute/subacute stroke, likely secondary to emboli. In view of her altered mental status and inability to protect her airway, she was intubated. She became hypotensive, requiring pressor support secondary to septic shock. EKG showed regularized atrial fibrillation, suggestive of a complete heart block with atrial fibrillation (Fig. 4). Therefore, a temporary transvenous pacing lead was inserted. Blood cultures were speciated as methicillin-sensitive Staphylococcus aureus, and antibiotics were switched to intravenous cefazolin.

**Fig. 5:**
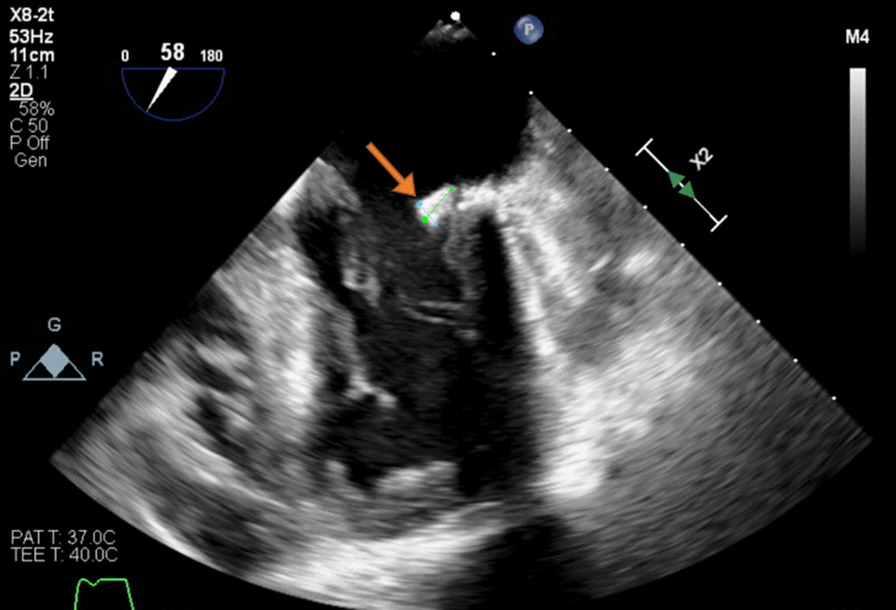
Transesophageal echocardiogram showing 0.76 cm × 0.50 cm vegetation (arrow) on the Posterior mitral valve leaflet

**Fig. 6:**
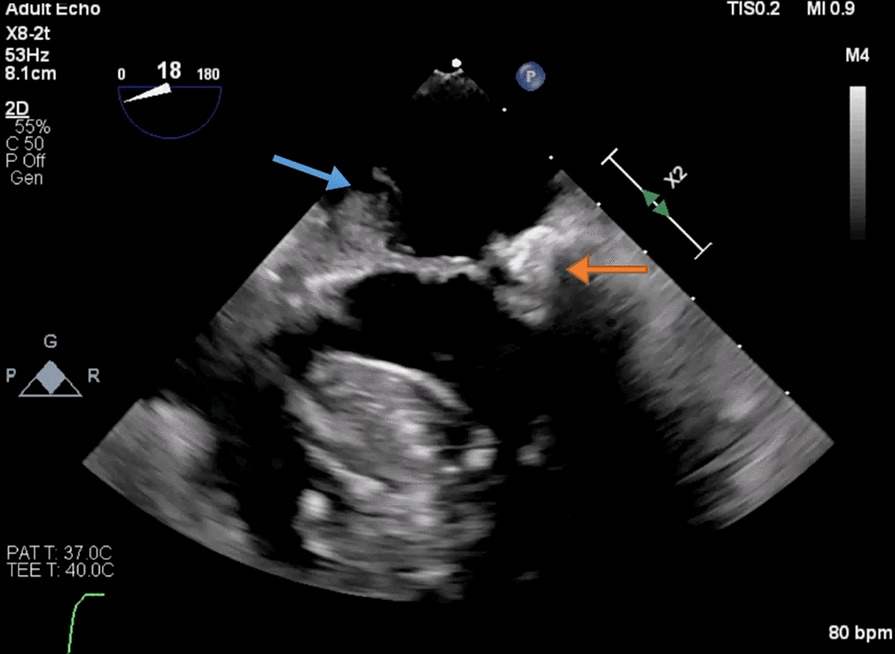
5 chamber view with vegetation on Posterior mitral valve leaflet (orange arrow) and large rounded echodensity along the aortomitral cushion (blue arrow)

**Fig. 7:**
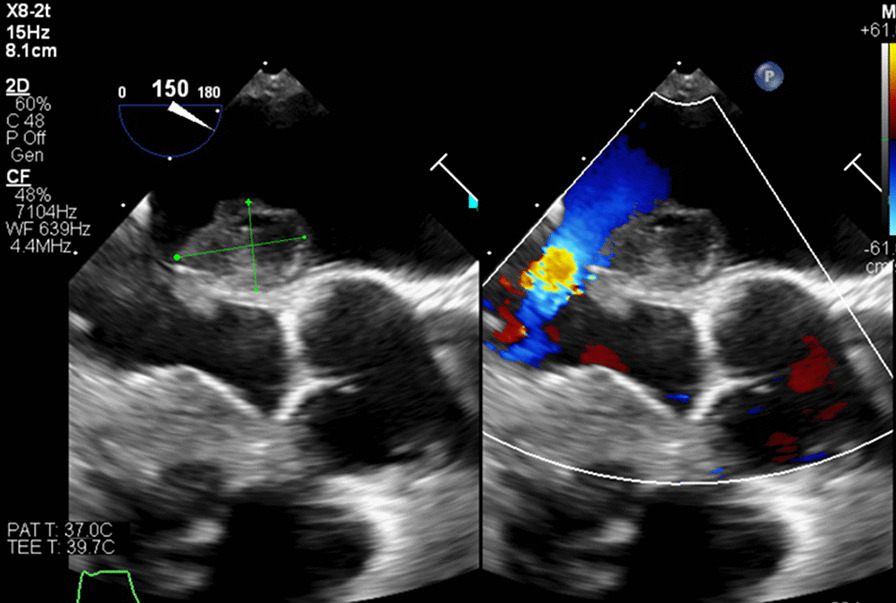
Transesophageal echocardiogram showing 1.9 cm × 1.3 cm polypoid echodensity on the atrial side of the anterior MVL/along the aortomitral cushion

**Fig. 8:**
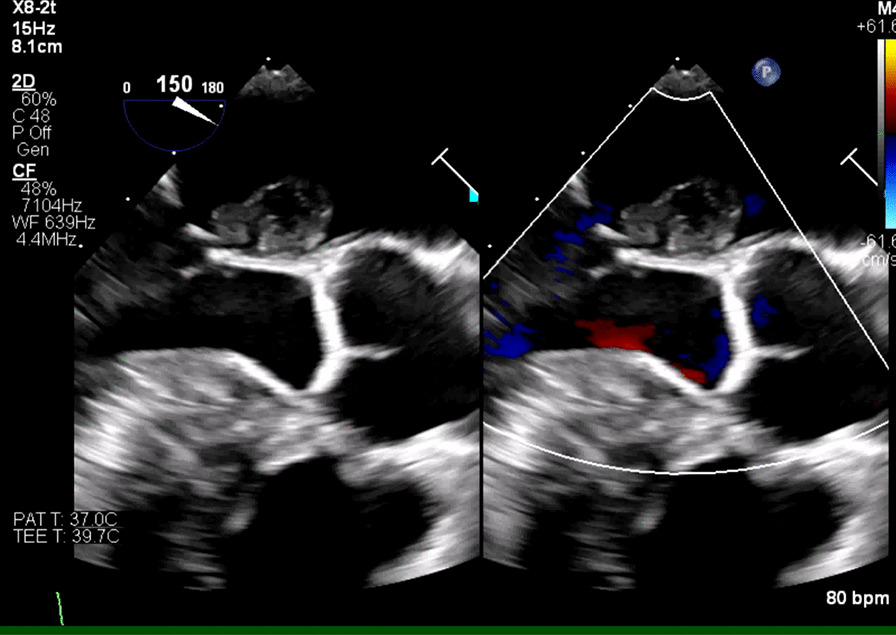
Transesophageal echocardiogram showing the polypoid echodensity on the atrial side of the Anterior mitral valve leaflet/along the aortomitral cushion

**Fig. 9:**
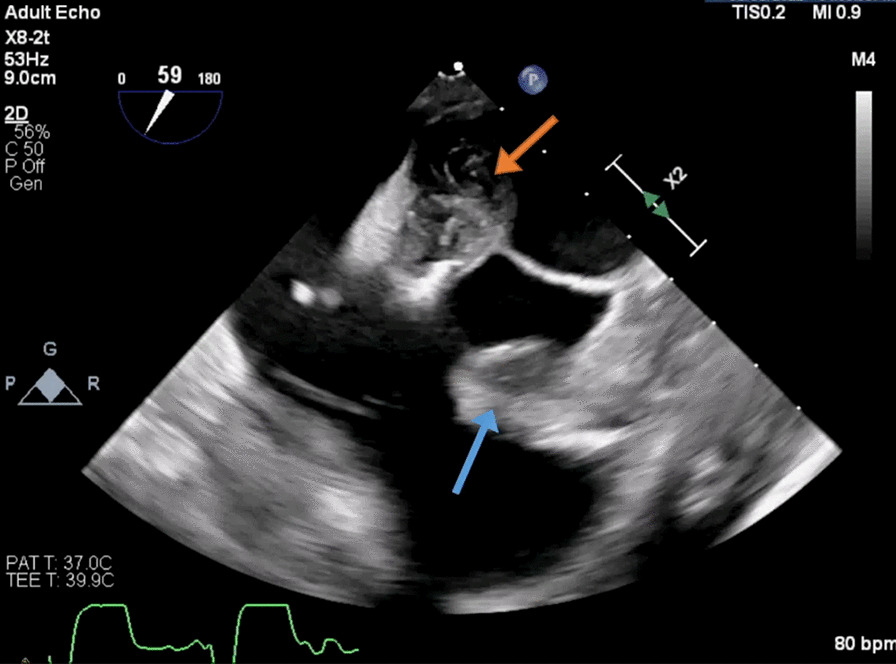
Abscesses along the aorto-mitral cushion (orange arrow) and within IVS extending from the aortic root (blue arrow)

**Fig. 10:**
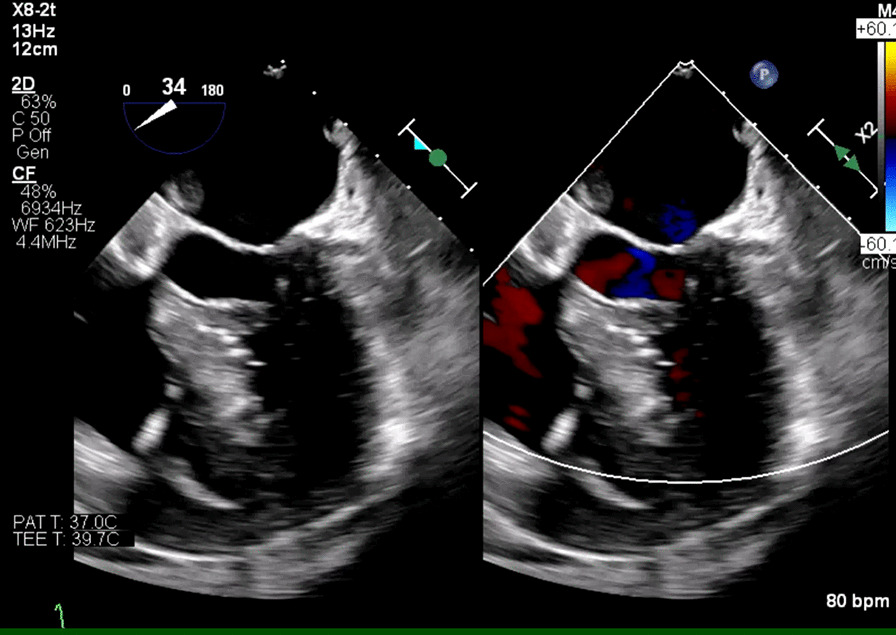
Pathologically thickened IVS with heterogeneous enhancement indicating interventricular septal abscess

On day 5, TEE revealed normal LV systolic function but found multiple echodensities, including a 0.76 cm × 0.50 cm vegetation seen on the PMVL (Figs. 5, 6) and a 1.9 cm × 1.3 cm polypoidal structure on the atrial side of the AMVL (Figs. 7, 8). In addition, there was a heterogenous extension of the echodensity from the aortic root, along the aorto-mitral cushion and into the IVS, which was significantly thickened, indicating IVSA (Figs. 9, 10). Mild mitral regurgitation and mild aortic regurgitation were noted along with a small pericardial effusion. Due to high mortality risk, she was not deemed to be a surgical candidate. Given her prohibitive surgical risk and poor prognosis, the family decided to change her goals of care to comfort measures only. Unfortunately, the patient died on day 6.

## Discussion

IE can progress into a cardiac abscess in 20 to 30% of cases, but the precise incidence of IVSA is unknown. IVSA is a rare but life-threatening complication of IE that can occur in developed countries in patients with predisposing risk factors such as prosthetic valves, intravenous drug use, deep penetrating wounds of the heart, infected cardiac implants, or immunocompromised states. IVSA usually presents with acute hemodynamic instability secondary to septic shock, with no definitive hallmark examination finding [5].

To the best of our knowledge, only a few case reports of IVSA have been published [6–13]. All of them presented with fever as the initial complaint, but our case is unique as the patient was afebrile on presentation. Three of the eight cases were in the pediatric age group. Our patient is also unique that she was in the eighth decade with no risk factors for IE. The atypical presentation in our patient could be attributed to aging or other associated comorbidities with an increased presence of chronic medical disease [14, 15].

The major complications of IVSA include cardiac tamponade, embolic stroke, multi-organ failure, and death, while minor complications include septal defects, pericarditis, and conduction blocks [6–13]. Most of them required open surgical incision and drainage of the abscess with a prolonged antibiotic regimen [4–8, 10–13], which is the standard of care in patients with IVSA.

## Conclusion

Due to the atypical presentation and low pretest probability, TEE as an initial testing modality for our patient was inappropriate, thus limiting early diagnosis [16]. As demonstrated in our case, rhythm abnormalities could be the only presenting feature of IVSA; hence we should consider performing a thorough infectious workup before proceeding with permanent pacemaker implantation for a complete heart block. Despite the aseptic presentation, it is worthwhile considering infectious etiology in patients with progressive heart block.

## Data Availability

All data are included in the abstract.

## Abbreviations

AMVL: Anterior mitral valve leaflet
CXR: Chest radiograph
EKG: Electrocardiogram
IE: Infective endocarditis
IVSA: Interventricular septal abscess
PMVL: Posterior mitral valve leaflet
TTE: Transthoracic echocardiogram
TEE: Transesophageal echocardiogram
